# Transcription factor with ambivalent role – *Ralstonia eutropha’s* PhaR is a repressor of the phasin-gene *phaP1* and an activator of *phaP3*

**DOI:** 10.1186/s12866-025-04628-7

**Published:** 2025-12-26

**Authors:** Paul Cornehl, Lara Santolin, Noa Clerc, Sebastian L. Riedel, Peter Neubauer, Matthias Gimpel

**Affiliations:** 1 Chair of Bioprocess Engineering, Technische Universität Berlin, Berlin, Germany; 2 Berliner Hochschule Für Technik, Environmental and Bioprocess Engineering Laboratory, Berlin, Germany

**Keywords:** *Ralstonia eutropha*, Polyhydroxyalkanoates, Gene regulation, Transcription factors, PhaR, Phasins

## Abstract

Polyhydroxyalkanoates (PHAs) serve as intracellular carbon and energy storage compounds in many prokaryotes. They accumulate in granules composed of a hydrophobic polymer core surrounded by a surface layer of proteins that regulate granule formation, stability, and degradation. Among these, the most abundant are low-molecular-weight, amphiphilic proteins known as phasins. In *Ralstonia eutropha* (*Cupriavidus necator*), one of the best-studied model organisms for PHA biosynthesis, the phasin PhaP1 controls the surface-to-volume ratio of the granule. Under non-accumulating conditions, the transcriptional repressor PhaR negatively regulates *phaP1* expression. A similar PhaR-mediated repression has also been proposed for another phasin gene, *phaP3*, though direct evidence has so far been lacking. In this study, the *phaP3* transcription start site was determined and three putative PhaR binding sites upstream and within the *phaP3* gene identified. Electrophoretic mobility shift assays confirmed PhaR binding to all three sites. Strikingly, β-galactosidase reporter-gene assays revealed that, unlike its repressive effect on *phaP1*, PhaR activates *phaP3* expression. Our findings provide the first direct evidence of the dual regulatory role of PhaR in *R. eutropha*, functioning as a repressor and activator of different phasin encoding genes. This suggests distinct physiological roles for PhaP1 and PhaP3 in PHA granule formation and turnover, further underscoring the complexity of the regulation of PHA metabolism.

**Supplementary Information:**

The online version contains supplementary material available at 10.1186/s12866-025-04628-7.

## Introduction

Approximately 40% of all prokaryotic strains, including both bacteria and archaea, naturally produce polyhydroxyalkanoates (PHAs) as intracellular carbon and energy storage compounds [12]. Owing to their biobased nature, versatile thermoplastic properties and biodegradability in common natural environments, PHAs are gaining traction in commercial markets as sustainable plastic alternatives [15, 18, 36]. PHAs are stored as water-insoluble, granule-like inclusions consisting of a polymer core surrounded by a surface layer of several PHA granule-associated proteins (PGAPs) [11]. Among these, the most abundant are low-molecular-weight, amphiphilic proteins known as phasins. In the best studied PHA producer *Ralstonia eutropha* (also known as *Cupriavidus necator*), eight phasins (PhaP1-PhaP8) have been identified [22, 23, 26, 33, 39]. The genes are scattered throughout both chromosomes and the megaplasmid (Fig. S1). The major phasin, PhaP1, controls the surface-to-volume ratio of the granule. Deletion of the *phaP1* gene results in cells producing less PHA in the form of a single big granule, whereas overexpression leads to the formation of numerous unusually small granules [39]. Due to significant lower expression levels of all other phasin-genes in comparison to *phaP1*, their deletion mutants do not show substantial phenotypic differences regarding granule number or size in comparison to the wild-type and therefore their function remains obscure [13, 22, 23]. Expression of *phaP1* is tightly regulated by the transcriptional repressor PhaR, which binds to the *phaP1* promoter region under non-PHA-accumulating conditions. PhaR does not belong to any of the well-characterized transcription factor families but contains an N-terminal helix–turn–helix (HTH) DNA-binding motif that is conserved among homologous proteins in other PHA-accumulating microorganisms [14]. During PHA synthesis, PhaR is sequestered to the nascent granule, thereby relieving repression of *phaP1,* whereas towards the end of PHA synthesis, the PHA granule, that is covered with phasins, can no longer accommodate PhaR, and the repression is again restituted [25, 40]. Since PhaR has also been shown to bind the upstream region of the phasin gene *phaP3* [27], a similar PhaR-mediated repression mechanism has been assumed for *phaP3* [11, 17, 21, 28, 29]. However, direct evidence of PhaR-mediated repression of *phaP3* expression has never been published. Intriguingly, several studies have reported distinct expression patterns of *phaP1* and *phaP3* under PHA-accumulating conditions: while *phaP1* is upregulated, as expected from relief of PhaR-mediated repression, *phaP3* expression is paradoxically downregulated by up to threefold [2, 21, 35]. Adding to this discrepancy, the positions of the PhaR binding sites differ significantly between both genes. At the *phaP1* gene, PhaR binds around the σ^70^ promoter of *phaP1* [25], whereas its binding site in *phaP3* is located within the coding region [27]. These observations suggest that, contrary to the previously assumed mechanism, *R. eutropha’s* PhaR might function as a transcription factor with an ambivalent role – acting as a repressor of *phaP1* while serving as an activator of *phaP3.*

To clarify the regulatory role of PhaR in *phaP3* expression, this study employed in vivo β-galactosidase reporter gene assays while also identifying the previously unknown transcription start site (TSS) for *phaP3* and verifying PhaR binding to its in silico predicted binding sites by electrophoretic mobility shift assays (EMSAs).

## Materials and Methods

### Bacterial strains and media

*Escherichia coli* DH5α [9] was used for plasmid propagation and as host for reporter gene assays, *E. coli* BL21 Gold (Agilent) was used for heterologous production of PhaR. All used strains are listed in Table S1. Transformation, plasmid propagation and heterologous PhaR production media have been described previously [30].

### Construction of PhaR production plasmids

The *phaR* gene was PCR amplified using primer pair MG0432/MG0433 (all used oligonucleotides are listed in Table S3) and chromosomal DNA from *R. eutropha H16* as template. The fragment was digested with BamHI and HindIII, and ligated into the corresponding pGW3 vector (Gimpel, unpublished, all used plasmids are listed in Table S2), yielding plasmid pGW3_PhaR and allowing IPTG inducible production of N-terminally His-tagged PhaR. The correctness of all inserts was verified by sequencing (see Supplementary data 1).

### Construction of reporter gene fusions

The previously constructed plasmid pGK_PphaP1_LacZ [30] served as template for a PCR with primer pair MG0310/MG0437 to amplify a 270 bp *phaP1* promoter fragment with an extended downstream region (−250 to + 20 relative to the TSS). The fragment was digested with EcoRI and BamHI and subsequently ligated into the corresponding pGK_LacZ vector (Gimpel, unpublished), yielding plasmid pGK_PphaP1.1_LacZ. Similarly, PCR with primer pair MG0435/MG0436 and chromosomal *R. eutropha* DNA was used to amplify a 390 bp fragment covering the *phaP3* promoter region (−276 to + 112 relative to the TSS). The fragment was digested with EcoRI and BamHI and ligated into pGK_LacZ cut with the same enzymes. The resulting plasmid pGK_Pphap3_LacZ contains a transcriptional *lacZ* fusion under control of the *phaP3* promoter. The correctness of all inserts was verified by sequencing (see Supplementary data 1).

### Prediction of PhaR binding sites

Putative palindromic PhaR binding sites in the promoter regions of phasin encoding genes (*phaP*1 and *phaP3*) were predicted manually. Sequences of phasin genes were obtained from the NCBI data bank (Accession number: AM260479). Based on the binding sites at *phaP1* and *phaP3* published by Pötter [27], the palindromic motif YTKNTGCRNYGCANMAR was deduced and used to search for putative additional PhaR binding sites in the regions 500 nt upstream and 200 nt downstream of the translation start of the phasin genes *phaP1* and *phaP3*. Sequences with a maximum of three mismatches from the consensus sequence were considered positive.

### Determination of the transcription start site of *phaP3*

The transcription start site of the *phaP3* gene was determined using adaptor- and radioactivity-free identification of transcription start sites (ARF-TSS) [38]. First, *R. eutropha* H16 was cultivated under non PHA producing conditions as described recently [30] and 500 µl samples were taken and flash frozen in liquid N_2_. Total RNA was purified using the Total RNA Mini Kit (A&A Biotechnology) according to the manufacturer’s instructions. Following elution of the RNA with 100 µl double-distilled water (ddH_2_O), the RNA was treated with DNase I (12 µl 10 × DNase I buffer, 8 µl DNase I (250 µg mL^−1^), AppliChem) for 10 min at 37 °C. Subsequently, the RNA was extracted by mixing with 80 µL ddH_2_O and 150 µL phenol. After centrifugation, the RNA containing the aqueous phase was transferred into 150 µL chloroform to remove residual phenol. After a 2nd washing step, the RNA was precipitated by addition of 1 mL 96% EtOH, 40 µL 3 M sodium acetate and 10 µL 10 g L^−1^ glycogen. After incubation at −20 °C for 1 h, the precipitation reaction was centrifuged for 10 min at 4 °C and 13.000 rpm. The pellet was washed with 200 µL 80% EtOH and finally resuspended in 20 µL ddH_2_O. The concentration of the purified total RNA was determined spectrophotometrically (NanoDrop One, Thermo Scientific). The production of cDNA was performed with SuperScript III reverse transcriptase (Invitrogen) according to the manufacturer's instructions. MG0448 phosphorylated at the 5' end was used as primer for cDNA synthesis. The cDNA was treated with RNase H (Invitrogen) for 20 min at 37 °C and afterwards purified by phenol/chloroform extraction and subsequent ethanol precipitation as described above. The cDNA was circularized using T4 RNA Ligase (New England Biolabs). The ligation reaction was performed at 25 °C overnight. The ligated cDNA was used as template for a PCR with primer pair MG0474/MG0475, the resulting fragments were digested with HindIII and BamHI, cloned into a pUC19 vector cut with the same restriction enzymes and subsequently sequenced to identify the TSS (Fig. S2, Supplementary data 2).

### Protein purification

His-tagged PhaR was purified from *E. coli* BL21 Gold containing the expression plasmid pGW3_PhaR. The cells were cultivated in a glucose releasing instant fed-batch medium (EnPressoB) according to the manufacturer’s instructions (EnPressoB GmbH, Germany). 24 h after induction with 0.1 mM IPTG to start PhaR production, cells were harvested by centrifugation. The pellets were stored at −20 °C until further use. For protein purification cell pellets were resuspended in lysis buffer (300 mM NaCl, 50 mM Na-phosphate, 10 mM imidazole, pH 8.0) supplemented with 0.1 mM protease inhibitor PMSF, 0.6 mg mL^−1^ DNaseI and 1 mg mL^−1^ lysozyme and subjected to sonification. The protein supernatant was subjected to affinity purification using Ni–NTA agarose columns. The columns were washed with five column volumes (CV) washing buffer (lysis buffer with 20 mM imidazole) and eluted six times with 0.5 CV elution buffer (lysis buffer with 200 mM imidazole). The protein concentration was determined spectrophotometrically (NanoDrop One, Thermo Scientific) and by SDS-PAGE as described recently [7].

### β-galactosidase reporter gene assay

*E. coli* DH5α was sequentially transformed with pGK_LacZ derivatives for *lacZ* gene expression under the control of the *phaP1* or *phaP3* promoter and pGW5_PhaR. Cultivation conditions and assay procedure have been described recently [30]. In brief, cells were cultured in square shaped 24 deep-well plates (Duetz-MTPS, Adolf Kühner AG) filled with 3 mL TY containing 1 mM MgSO4 for 6 h. PhaR production was induced after 2 h by addition of 200 µM IPTG. Samples normalized to 625 µL of culture at OD_600_ = 1 were harvested, disrupted and the LacZ activity in the supernatant measured, using ortho-nitrophenyl beta-D-galactopyranoside as a substrate analysed by measuring *A*_420_ as described recently [30]. β-galactosidase activity was calculated according to Eq. 1.

1 $${Activity}_{\beta -Galactosidase}(Miller Units (MU))=\frac{1500 \times {A}_{420}}{0.5 \times {Incubation time}_{(\mathrm{min})}}$$

### Electrophoretic mobility shift assays (EMSA)

DNA fragments covering the putative PhaR binding sites in P_*phaP3*_ were amplified by PCR using primer pairs MG0435/MG0451 (BS1, *phaP3*), MG0434/MG0457 (BS2, *phaP3*), MG0456/MG0453 (BS3, *phaP3*), MG0435/MG0457 (BS1/2, *phaP3*) and MG0434/MG0453 (BS2/3, *phaP3*) and chromosomal DNA from *R. eutropha* as template. Primer pair MG0514/MG0515 and plasmid pUC19 as template were used to generate a control fragment. A 5’fluorescein amidite (FAM) label on the downstream primers tagged all fragments fluorescently (Fig. S2). For EMSAs, a serial dilution of purified PhaR (0–250 ng) was incubated with 5 µL master mix (containing 1.75 µL 10 × TBE buffer, 0.25 µL 10 mg mL^−1^ herring sperm DNA and 3 µL labelled DNA (~ 75 ng)) for 30 min at 37 °C. After incubation for 30 min at 37 °C, 2.5 µL loading dye (40% glycerol, 250 mM Tris–HCl, pH 7.5) were added and the samples were loaded onto 7.5% (37.5:1) native polyacrylamide gels. The gels were analyzed using a BioRad ChemiDoc Go (BioRad, Germany).

## Results and Discussion

### Transcription of *phaP3* starts 23 bp upstream of the translation initiation site

**Fig. 1:**
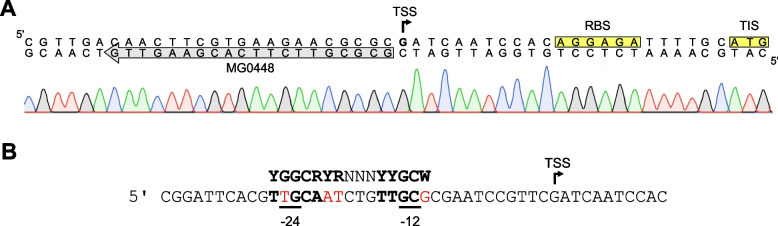
Identification of the *phaP3* transcription start site in *R. eutropha* H16 using ARF-TSS. **A**: A representative sequencing result used for determination of the *phaP3* TSS. The 3’ phosphorylated primer MG0448 used for cDNA synthesis during ARF-TSS is indicated as grey arrow. Ribosome binding site (RBS) and translation initiation site (TIS) are highlighted in yellow. The identified TSS is indicated by a bent arrow. **B**: Identification of a σ^N^-like promoter upstream of the *phap3* TSS (bent arrow). Nucleotides matching the σ^N^ -consensus (YGGCRYR(N)_4±1_YYGCW) are highlighted in bolt, whereas mismatches are shown in red. The putative −24/−12 boxes are underlined

Previous studies have stated that PhaR is a repressor of phasin-encoding genes in *R. eutropha* [25, 27]. While an almost perfect σ^70^-like promoter was previously identified for *phaP1* [25], several putative promoters dependent on alternative sigma factors involved in general stress (σ^S^) and nitrogen metabolism (σ^N^) have been predicted for *phaP2* and *phaP4-phaP7* [30]. In contrast, no promoter for the *phaP3* gene could be predicted, making it impossible to assess the relative position of the PhaR binding sites to the promoter and thus to estimate their regulatory effect. To address this issue we aimed at identification of the transcription start site (TSS) and the corresponding promoter of the *phaP3* gene. Using ARF-TSS [38], a G residue 23 bp upstream of the translation initiation site (TIS) was identified as TSS (Fig. 1A). Sequence analysis revealed a region located 12 bp upstream of the newly identified TSS that displayed homology (08/12) with the eubacterial σ^N^-promoter consensus sequence [1] (Fig. 1B). Phasin gene expression under control of a nitrogen dependent promoter is consistent with the fact that PHA polymer accumulation is triggered by various stresses, including nitrogen starvation [24, 37].

### In silico predicted PhaR binding sites in *phaP1* and *phaP3* differ in position relative to the TSS

**Fig. 2:**
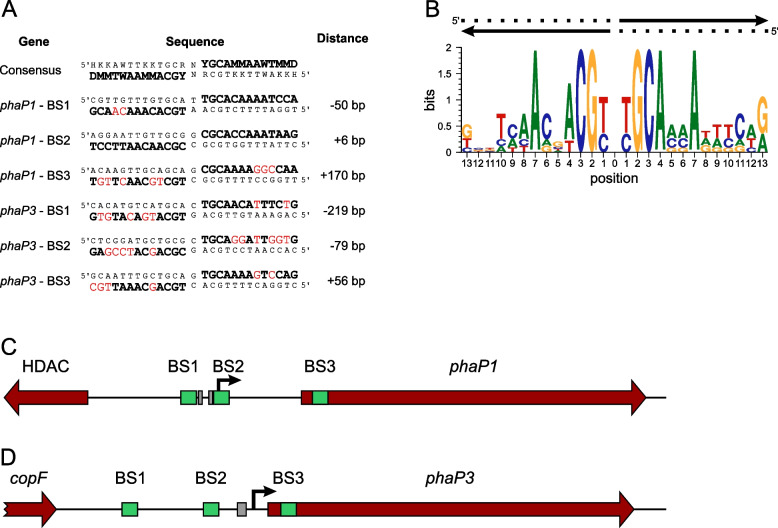
In silico prediction of PhaR binding sites in *phaP1* and *phaP3* sequences in *R. eutropha* H16. **A:** Alignment of putative PhaR binding sites. Deviations from the consensus sequence according to [25] are highlighted in red. The distance of the central bp to the TSS is indicated. **B:** Sequence logo of the putative PhaR binding sites. The logo was created using SRPlot [34]. DNA strands are indicated as black arrow. **C:** Organization of the PhaR binding sites at P_*phaP1*_ and **D:** P_*phaP3*_. The open reading frames are indicated with red arrows, TSS for *phaP1* and *phaP3* are indicated with bent black arrows, in silico predicted Binding Sites (BSs) and promoter boxes are highlighted in green and grey, respectively

Prior to this study, two regions upstream of the *phaP1* coding region had been identified as PhaR binding sites using DNase I Footprinting [25]. These sites are located directly upstream of the −35 box (BS1) and around the TSS (BS2) (Fig. 2C). Three 12 bp sequences – one at BS1 and two at BS2 – were proposed as binding motifs based on sequence alignment and consensus analysis [26]. Similarly, a PhaR BS containing a single 12 bp binding motif had been identified within the coding region for *phaP3* (BS3, Fig. 2D) [27]. However, typically DNA binding transcription factors form multimers that recognize palindromic binding sites [16]. Such a palindromic arrangement of the PhaR binding motifs could only be shown in BS2 at the *phaP1* promoter. Thus, we first looked at the sequences adjacent to the two individual PhaR binding motifs at P_*phaP1*_ and P_*phaP3*_. Interestingly, significant similarities to the consensus sequence were also found for these regions (Fig. S3). A comparison of the PhaR binding sites shows that in all cases they are degenerate palindromes with a central single nucleotide spacer and a particularly highly conserved central region (Fig. 2A). Based on the palindromic architecture of the PhaR binding sites, we searched in silico for additional binding sites in the *phaP1* and *phaP3* genes. Two additional PhaR BSs were predicted approximately 200 bp (BS1) and 70 bp (BS2) upstream of the *phaP3* TSS, respectively (Fig. 2A and Fig. 2D). Moreover, a putative BS3 was predicted within the *phaP1* ORF (Fig. 2A and Fig. 2C). The comparison of the predicted binding sites illustrates that, starting from the centre of the binding site, positions 1–4 and 7 are almost perfectly conserved (Fig. 2B). In contrast, the other positions show hardly any conservation. Although some of the newly predicted binding sites deviate significantly from the consensus sequence according to Pötter [25], the functionality of the binding sites cannot be ruled out. On the one hand, the deviations are almost exclusively located within poorly conserved positions and on the other hand, the consensus sequence is based on only four sequences and is therefore not particular robust.

An obvious difference is the relative position to the promoter of the PhaR binding sites at *phaP1* and *phaP3*. While BS1 and BS2 at *phaP1* are adjacent to the −35 box and the TSS, respectively, BS1 and BS2 at *phaP3* are clearly upstream of the promoter. The observed differences in the positions of PhaR binding sites relative to the TSS in *phaP1* and *phaP3* suggest that these genes may be subject to different regulatory mechanisms [3, 4]. In contrast to the positions of the PhaR binding sites on *phaP1*, which are typical for a repressor that acts via steric hindrance, the binding sites on *phaP3* are too far upstream of the promoter for steric hindrance and are more likely to bind a transcriptional activator. Interestingly, for both genes a third putative binding site located in the ORF was predicted. The organization of the binding sites at *phaP1* is very similar to the organization of the LacI binding sites at the *E. coli* Lac operon. Likewise, LacI has a primary binding site overlapping the transcription start and two auxiliary operators upstream of the −35 box and within the ORF, respectively. Interaction with the auxiliary operators stabilizes the binding of tetrameric LacI to the main operator and thus enhances repression in the absence of inducer [19, 20]. A similar function of the three PhaR binding sites on *phaP1* is conceivable. Consequently, BS3 in *phaP3* could also be an auxiliary operator that stabilizes the PhaR binding to the assumed major binding site BS2.

### PhaR binds *phaP3* gene at three binding sites

**Fig. 3:**
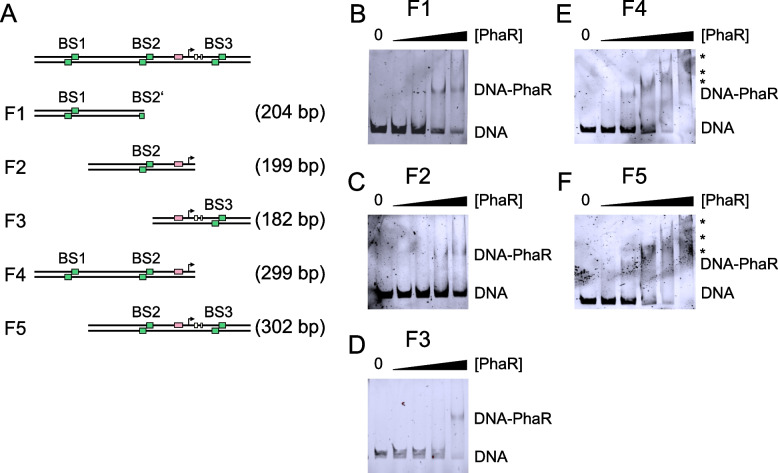
Confirmation of PhaR binding to three binding sites in *phaP3* by in vitro Electrophoretic Mobility Shift Assays (EMSAs). **A**: Schematic representation of DNA fragments (F1-5) used for EMSAs. Green, white, black and pink boxes represent the in silico predicted PhaR binding sites (BS), ribosome binding site, start codon, and promoter region, respectively. In addition to BS1, Fragment 1 contains a part of the left arm from BS2 at its 3’ end. The transcription start site is represented by a bent arrow. **B – F**: EMSAs performed with F1-5, respectively. Increasing amounts of PhaR (0, 15.6, 31.2, 62.5, 125 ng) were used in B-D while an additional 250 ng was used in E and F. Shifts marked with an * are a result of the formation of supramolecular complexes

To validate the in silico predicted PhaR BSs in *phaP3*, PhaR was heterologously produced in *E. coli* and purified via a His-tag for use in in vitro Electrophoretic Mobility Shift Assays (EMSAs). Six distinct FAM-labelled DNA fragments were synthesized for this purpose: F1-F3 each covering a single BS (BS1-BS3, respectively) and F4 and F5 covering two adjacent BSs (BS1 + BS2 and BS2 + BS3, respectively) (Fig. 3A) and a 200 bp pUC19 fragment as specificity control. EMSAs confirmed the binding of PhaR to each single in silico predicted BS (Fig. 3B-D), whereas no binding of PhaR to the control fragment was detectable (Fig. S4). With increasing PhaR concentrations, a decreasing amount of free DNA was observed as DNA-PhaR complexes were formed. BS1 was most strongly bound by PhaR and therefore is probably the main BS. In contrast, the binding of PhaR to BS2 or BS3 is significantly weaker (Fig. 3. B-D). When DNA fragments covering two adjacent BSs were tested, a “runup” of the shift was observed with increasing PhaR concentrations, which is a result of supramolecular complexes being formed as more than one PhaR molecule binds the DNA. Again, the fragment covering BS1 is more strongly bound by PhaR further corroborating that BS1 is the main BS (Fig. 3E). Moreover, in both cases lower PhaR concentrations are required to shift the DNA if more than one binding site is present indicating a cooperative effect of the two binding sites (Fig. 3B-D vs. Fig. 3E-F). Although EMSAs had already been employed to confirm specific binding of PhaR to the *phaP3* promoter region [27] only an interaction with the fragment covering BS3 could be detected [27]. However, in this previous study, various DNA fragments were simultaneously incubated with PhaR, resulting in potential competition for binding complicating the interpretation of shift patterns [10].

### PhaR is a transcriptional activator of *phaP3*

**Fig. 4:**
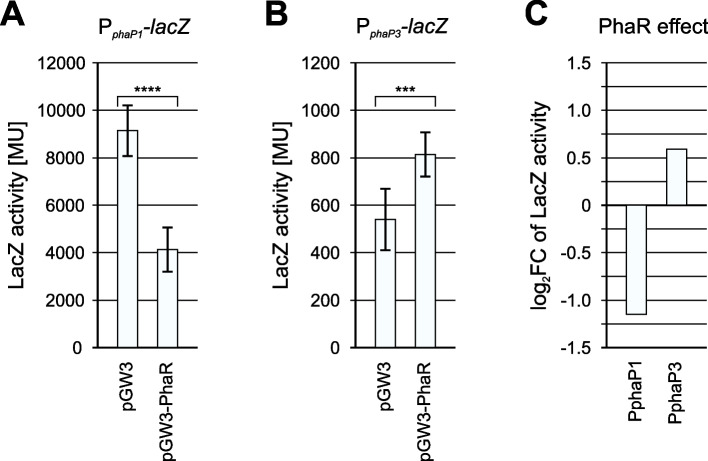
Impact of PhaR on expression from *Ralstonia eutropha* H16 P_*phaP1*_ and P_*phaP3*_ promoters in vivo. **A**: Impact on expression from P_*phaP1*_ promoter. **B**: Impact on expression from P*phaP3* promoter. The measured β-galactosidase activity when co-expressing PhaR is displayed in contrast to a control with an empty plasmid for PhaR expression. Error bars represent the standard deviation of 4 to 6 independent biological replicates. A two-sided Welch’s t-test was performed to assess the statistical significance of the observed effects with p-values of 6.4 × 10⁻⁶ for P_*phaP1*_ (p < 0.0001;****) and 7.0 × 10⁻^4^ for P_*phaP3*_ (p < 0.001; ***) **C**: The measured β-galactosidase activity is presented as a log_2_-fold change against the control with an empty plasmid for PhaR

Finally, the impact of PhaR on expression from *R eutropha* phasin promoters P_*phaP1*_ and P_*phaP3*_ was investigated by in vivo β-galactosidase reporter-gene assays performed heterologously in *E. coli* with overexpression of PhaR (Fig. 4). In line with previous studies [25, 40], co-expression of PhaR imparted a twofold repression of expression from P_*phaP1*_ (Fig. 4A,C). Confirming our working hypothesis and contradicting earlier assumptions, co-expression of PhaR resulted in a 1.5-fold activation of expression from P_*phaP3*_ (Fig. 4B,C). Thus, this study provides direct evidence of the ambivalent role of the transcriptional factor PhaR on phasin expression in *R. eutropha*, functioning as a repressor of the major phasin gene *phaP1,* while activating *phaP3*. Although, reporter-gene assays in a heterologous host may not perfectly reproduce the conditions in the native organism – due to differences in the availability of sigma factors, cofactor concentrations, or the absence of PHA granules – which influence the regulatory dynamics and can lead to quantitative discrepancies, this methodology is well established, has previously been successfully applied to *R. eutropha* promoters [8, 30] and provides a robust and reproducible first indication of direct regulatory effects, which can be further validated in *R. eutropha* in the future.

The large number of phasins (PhaP1-8) in *R. eutropha* suggests that they do not only have a redundant function in stabilizing PHA granules, but rather distinct functions. Different phasins may be required at different stages during PHA synthesis, storage, and/or degradation to control the formation and breakdown of granules. Various studies have provided evidence suggesting that phasins can interact with depolymerases to prevent or activate PHA degradation [6, 13, 22, 32]. Although the exact function of PhaP3 has not yet been elucidated, transcriptome data [2] suggest that PhaP3, unlike PhaP1, has a function at a very early stage during PHA synthesis, potentially during the initiation of PHA synthesis. Before substantial PHA production begins, activation of the weak *phaP3* promoter may allow the production of the necessary amount of PhaP3 required for the initiation of PHA synthesis. At this stage, PhaR binding to P_*phaP1*_ prevents the production of PhaP1, which is not yet needed. As PHA synthesis progresses, PhaR is sequestered by the nascent PHA leading to a halt in activation of expression of the now no longer needed PhaP3, and in turn, relieves the repression of *phaP1*.

Overall, this study highlights once more the complexity of PHA regulation in *R. eutropha* and suggest that PhaP1 and PhaP3 may have distinct functions, as their expression is differentially regulated.

## Conclusion

**Fig. 5:**
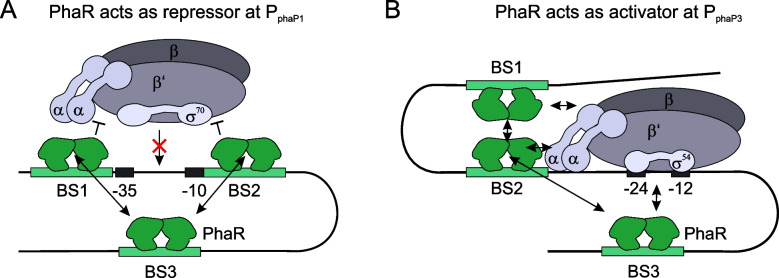
Working model of PhaR-mediated transcriptional regulation of **A:** *phaP1* and **B:** *phaP3*. Binding sites (BS), and promoter −35/−10 and −24/−12 boxes are depicted as green and black boxes. PhaR dimers are depicted in green attached to each BS. RNA polymerase α, β, β’ and σ subunits are depicted in grey

Our results suggest that the function of PhaR in the regulation of phasin gene expression in *R. eutropha* H16 is much more complex than previously anticipated. At both the *phaP1* and *phaP3* promoter, three palindromic PhaR BSs with two binding motifs each could be identified which are most likely bound by PhaR dimers. Reporter gene assays confirmed the previous hypothesis that PhaR indeed acts as a repressor of the *phaP1* gene. The repressive function is consistent with the location of the PhaR BSs. BS1 and BS2 are located in the direct vicinity of the −35 and −10 boxes, respectively. Inhibition of transcription by steric hindrance of RNA polymerase (RNAP) binding is therefore likely (Fig. 5A). A PhaR dimer that binds to BS3 located further downstream might stabilize the binding of PhaR to BS1 or BS2 by dimer-dimer interaction and thus enhance repression. Such a function has already been shown for the repression of the *lac* promoter by *E. coli* LacI [19, 20]. In contrast, we were able to show that PhaR has a different role at the *phaP3* promoter, where it serves as an activator. This different function is already reflected in the position of the BSs. BS2 is located upstream of the promoter region where a PhaR dimer could stabilize RNAP binding to the promoter region through contacts with the α-subunit (Fig. 5B). Analogous to the *phaP1* promoter, bound PhaR at BS1 or BS3 could stabilize PhaR binding to BS2 through dimer-dimer interaction and subsequent loop formation. Forming such a loop to stabilize an activator is also found in the regulation of the *E. coli* arabinose operon by AraC [31]. Also, PhaR at BS1 or BS3 could function through additional contacts with the RNAP or the sigma factor. In contrast to $\sigma$ ^70^, σ^54^ is dependent on activator support for the transition from the closed to the open RNAP complex. Typically, this is accomplished by the ATPase activity of the activator [5]. Although such ATPase activity has not yet been shown for PhaR, the loop formation between BS2 and BS3 might generate a strong DNA bend that could lead to the opening of the DNA double strand in the promoter region and thus enable the transition to the open complex.

Future research will focus on elucidating the role of PhaR at the *phaP3* promoter in vivo in *R. eutropha*. It is tempting to speculate whether PhaR is also involved in the regulation of other phasins or PHA depolymerases in *R. eutropha,* and to what extent genetic engineering could harness PhaR – both as an activator and as a repressor – to modulate additional genes involved in PHA metabolism. Such targeted regulation by a single transcriptional factor could provide a powerful tool for optimizing biotechnological PHA production in *R. eutropha*.

## Supplementary Information

Supplementary Material 1.

## Data Availability

The datasets supporting the conclusions of this article are included within the article and its additional files.
