# Supplementary material for: Transcription factor with ambivalent role – Ralstonia eutropha’s PhaR is a repressor of the phasin-gene phaP1 and an activator of phaP3
Source: BMC Microbiol. 2025 Dec 26;26:61. doi: 10.1186/s12866-025-04628-7 (PMC12849132; doi:10.1186/s12866-025-04628-7)
Supplement: Supplementary file 1 — Supplementary Material 1. [file 12866_2025_4628_MOESM1_ESM.zip › Cornehl and Santolin et al. - Supplementary material - Revision 1.docx]

Supporting Information

**Transcription Factor with Ambivalent Role –** ***Ralstonia eutropha’ s* PhaR is a Repressor of the Phasin-Gene *phaP1* and an Activator of *phaP3***

Paul Cornehl^1,#^, Lara Santolin^1,#^, Noa Clerc^1^, Sebastian L. Riedel^2^, Peter Neubauer^1^, Matthias Gimpel^1,*^

^1^ Technische Universität Berlin, Chair of Bioprocess Engineering, Berlin, Germany

^2^ Berliner Hochschule für Technik, Environmental and Bioprocess Engineering Laboratory, Berlin, Germany

^#^ **Contributed equally to this work and share first authorship**

*** Correspondence:**

Matthias Gimpel

matthias.gimpel@tu-berlin.de

***Table S1| Bacterial strains used in this study***

| **Strain** | **Genotype** | **Reference** |
| --- | --- | --- |
| *Ralstonia eutropha H16* | Wild-type strain, Gm resistant | ATCC 17699 |
| *Escherichia coli* DH5α | F^-^ *endA1 glnV44 thi-1 recA1 relA1 gyrA96 deoR*  *nupG purB20 φ80dlacZΔM15 Δ(lacZYA-argF)U169 hsdR17(r_K_^–^m_K_^+^) λ-* | (Hanahan, 1985) |
| *E. coli* BL21 Gold | F^-^*ompT hsdS(r_B–_ m_B–)_ dcm^+^ Tet^R^ gal endA The* | Agilent, Waldbronn |

***Table S2 | Plasmids used in this study***

| **Plasmid** | **Description** | **Reference** |
| --- | --- | --- |
| pUC19 | *Escherichia coli* expression vector, Amp^R^ | (Yanisch-Perron, et al., 1985) |
| pGW3 | *E. coli* expression vector, P*_Lac_*, His-tag, *Bacillus subtilis* BsrF terminator, ColE1 ori, *parB*, Amp^R^ | Gimpel, unpublished |
| pGW3_PhaR | As pGW3, with *Ralstonia eutropha phaR* gene | This work |
| pGK-LacZ | *E. coli/B. subtilis* shuttle vector, pWV01 ori, promoterless *lacZ* gene, Spec^R^ | Gimpel, unpublished |
| pGK-PphaP1-lacZ | As pGK-LacZ, with *R. eutropha* P*_phaP1_* | (Santolin et al., 2024) |
| pGK-PphaP1.1-lacZ | As pGK-LacZ, with *R. eutropha* P*_phaP1_* with extended downstream region | This work |
| pGK-PphaP3-lacZ | As pGK-LacZ, with *R. eutropha* P*_phaP3_* | This work |

***Table S3: Oligonucleotides used in this study***

**Name Sequence purpose**

MG0272 5’ TGG CGT CAC AGC CGC TCC CGT EMSA

MG0310 5’ ATC GAA TTC TGG TCG GCA TAG AAA GCG AGC C, P_phaP1_-Lac

MG0432 5’ ATC GGA TCC GCC ACG ACC AAA AAA GGC GC C, pGW-PhaR

MG0433 5’ ACT AAG CTT CAG CGT GCG GGA TAT GCG C, pgW-PhaR

MG0434 5’ ATC GAA TTC ACG ATG CAC TTT TAT TGT CCT EMSA

MG0435 5’ ATC GAA TTC TGT ATT CAG AGA TCC GTT CCA C, P_phaP3_-Lac, EMSA

MG0436 5’ ATC GGA TCC GCC GTC AAA AGC CAT GTT CGT C, P_phaP3_-Lac

MG0437 5’ ATC GGA TCC AAG GCG CAT TTC TTA TTT GGT GCG C, P_phaP1.1_-Lac

CCG CAA CAA TTC CTA TT

MG0448 5’ [Phos]-GCG CGT TCT TCA CGA AGT TG TSS

MG0449 5’ ATC AAG CTT CAT ATG CAA CCG CCT TCT CC TSS

MG0450 5’ ATC GAA TTC AAG CTT GTA CGA AAT CGC ATC GAA TSS

CA

MG0451 5’ [6FAM]-AGC ATC CGA GTG CGA ATC CTA GAG EMSA

MG0453 5’ [6FAM]-TCC GCC GTC AAA AGC CAT GTT CGT EMSA

MG0454 5’ TAT GCA CGC CAT GTG TAC GAA ATC TSS

MG0455 5’ CGA ACA CTT CAC GCA GGT CCT TTC TSS

MG0456 5’ GGC GCT GTC AAT CCG GAA GCC AA EMSA

MG0457 5’ [6FAM]-CTG TGG ATT GAT CGA ACG GAT TCG CGC EMSA

MG0463 5’ [6FAM]-CTT CAA GGT CTA GCA AAT TAA GCC EMSA

MG0514 5’ GTG TGA AAT ACC GCA CAG ATG EMSA

MG0515 5’ [6FAM]-ACT GGC CGT CGT TTT ACA ACG EMSA

M13-24R 5’ CGG ATA ACA ATT TCA CAC AGG S

M13rev2 5’ GAG TTA GCT CAC TCA TTA GG S

LacZ93 5’ CGC CAG CTG GCG AAA GGG S

__________________________________________________________________________________________

C, construction of plasmid followed by plasmid designation; EMSA, oligos for generation of PCR fragments used in EMSA; S, sequencing; TSS, oligos used for ARF-TSS

**Figure S1 | A**: Chromosomal organization of the eight phasin genes (*phaP1‑8*) and the *phaR* gene in *R. eutropha* H16. The genes of interest and their adjacent genes are shown in a true-to-scale scheme. The open reading frames are indicated by arrows (Blue: phasin genes; brown: *phaR* gene; yellow: genes encoding proteins involved in PHA metabolism; grey: other genes). The numbers between the arrows indicate the distance between two open reading frames, while the numbers on the left and right indicate the chromosomal position based on the *R. eutropha* genome (GenBank: AM260479.1). Apart from *phaP8* all other phasin genes and the *phaR* gene are isolated single genes. Their loci are scattered throughout the genome. *phaP1*, *phaP3*, *phaP8* and *phaR* are encoded on chromosome 1, *phaP4-7* are encoded on chromosome 2 and *phaP2* is encoded on the megaplasmid pHG1.

**Figure S2 |** Position of oligonucleotides used in ARF-TSS, generation of templates for EMSA and cloning of the *phaP3* promoter region for reporter gene assay. All oligonucleotides are indicated as black arrows. FAM-labels at MG0451, MG0453 and MG0457 are indicated by a green circle. The red circle at MG0448 indicates the 5’ phosphorylation. Bent arrow: transcription start site, green boxes: putative PhaR binding sites, red box: putative *phaP3* promoter, yellow boxes; RBS, start codon and stop codon.

**A**

**B**

**Figure S3 | A**: Alignment of putative single PhaR binding motifs. Deviations from the consensus sequence according to (Pötter et al., 2002) are highlighted in red. The bases that match the consensus sequence are marked in bold The absolute number of occurrences of a base per position is shown below the sequence alignment.. The coverage of the individual positions by the consensus sequence is indicated. **B**: Sequence Logo of the consensus motif based on the sequences shown in A.

**Figure S4 |** Confirmation of the specificity of PhaR binding by *in vitro* Electrophoretic Mobility Shift Assays (EMSAs). **A**: EMSA was performed with 204 bp fragment covering BSI at P*phaP3* (Fragment F1) and a 200 bp heterologous control fragment without any PhaR binding site obtained from plasmid pUC19, respectively. Increasing amounts of PhaR (0, 31.2, 62.5, 125 ng) were used. With increasing amounts of PhaR a shift is detectable with fragment F1 while no such shift is detectable with the control fragment.
